# Appetite and Gastrointestinal Hormone Response to a Gluten-Free Meal in Patients with Coeliac Disease

**DOI:** 10.3390/nu11010082

**Published:** 2019-01-03

**Authors:** Paola Vitaglione, Fabiana Zingone, Nicolina Virgilio, Carolina Ciacci

**Affiliations:** 1 Department of Agricultural Sciences, University of Naples “Federico II”, 80055 Portici, Italy; paola.vitaglione@unina.it (P.V.); nicolina.virgilio@unina.it (N.V.); 2 Department of Surgery, Oncology, and Gastroenterology, University of Padua, 35100 Padua, Italy; fabiana.zingone@unipd.it; 3 Department of Medicine, Surgery, and Dentistry, “Scuola Medica Salernitana” University of Salerno, 84084 Fisciano, Italy

**Keywords:** coeliac disease, appetite, ghrelin, glucagon-like peptide-1, gluten-free diet, ghrelin

## Abstract

Coeliac disease (CeD) is an immune-mediated inflammatory enteropathy triggered by the ingestion of gluten in genetically susceptible individuals. Gastrointestinal (GI) hormone response related to appetite and glucose metabolism is still under-investigated in patients with CeD. This study aimed at shedding light on the appetite sensations, glycaemia and hormone response induced by a complex meal in patients with coeliac disease. Twenty-two women with CeD, nine at the diagnosis (CeDD) and thirteen under a gluten-free diet (CeDGF), and ten healthy subjects (HS) were enrolled in a single day intervention study. All subjects consumed a test meal, recorded their appetite sensations, and blood was collected over three hours after meal consumption. The study found a lower decrease in hunger in CeDD compared to CeDGF and HS after meal intake. Data showed no difference of fullness and satiety between the groups. CeDD had lower insulin and glucose-dependent insulinotropic polypeptide (GIP) than CeDGF and HS. Both CeDD and CeDGF experienced a lower post-prandial response of glucose than HS. Data suggested that patients with CeD have an impaired glucose absorption after more than 12 months of gluten-free diet. Postprandial GIP may play a significant role in appetite cues and insulin response to a complex meal.

## 1. Introduction

The incidence of coeliac disease (CeD) has significantly increased over the recent decades especially among adults [1,2]. It is well known that a gluten-free diet (GFD) is the mainstay of treatment for CeD but a spectrum of disorders often coexists in patients with CeD [3] that are not completely solved by a GFD such as the gastrointestinal functional disorders [4]. Mounting evidence shows that some health conditions may even be worsened by GFD because it has been associated with increased risk of metabolic syndrome, obesity, and cardiovascular disease [3] which may be dramatic in CeD patients who are genetically predisposed to develop type 2 diabetes [5]. The increased risk of overweight or obesity was reported in patients with CeD who were normal weight or overweight and even experiencing cholesterol levels below the normal range at diagnosis [6,7,8,9]. This observation was associated with both the improved intestinal absorption induced by the GFD and to the unhealthy dietary behaviour adopted by CeD patients [10,11] who frequently have diets rich in lipids, sugars and proteins [12,13,14] as well as low in dietary fibre [12,13,15]. The abuse of commercial gluten-free (GF) products usually having a higher content of fats (mainly saturated) and lower amount of micronutrients than the same products with gluten may account for the overall nutritional inadequacy of GFD and the associated health effect [16,17,18]. Regarding the cardiovascular disease risk associated with the GFD, a recent systematic review of the intervention and observational studies focused on the effects of a GFD for at least 6 months on clinical markers of cardiometabolic risk, showed that GFD increased total cholesterol, high density lipoproteins, fasting glycaemia and body mass index [19]. Moreover, the authors reported that the main difficulty to determine the effect of the GFD on risk of disease is the absence of literature studies including a healthy control group which would also be useful to evaluate the adequacy of adopting a GFD from healthy people, that is a current dietary trend [18].

From a physiological perspective, long-term metabolic effect of diets results from fine homeostatic and non-homeostatic (reward) mechanisms taking place in the post-prandial state. Indeed, nutrient sensing along the gastrointestinal (GI) tract and hormonal response can modulate both food choice and frequency of eating by regulation of appetite sensations [20].

Glucose plays a metabolic role in the control of hunger, satiety and the regulation of body energy balance [21]. The glucostatic theory of appetite control suggests that reduced glucose utilisation in critical brain regions leads to perception and expression of hunger [22]. Therefore, pre-prandial hypoglycaemia, reduced availability of glucose for metabolism and a decreased level of body carbohydrate stores or liver glycogen are stimuli for increased food intake [21]. Insulin and the major incretins, glucose-dependent insulinotropic polypeptide (GIP) and glucagon-like peptide-1 (GLP-1) with their glucoregulatory functions play a key role on appetite [23]. GIP dose-dependently controls postprandial glucose mainly through an increased insulin release, while GLP-1 also acts by an initial slowing of gastric emptying [23]. As a consequence, once L-cells of distal ileum and gut sense fats and carbohydrates, they stimulate GLP-1 that inhibits the acid secretion in the stomach and decelerates the gastric emptying by inducing an anorexigenic effect [24]. On the contrary ghrelin, that is mainly secreted by oxyntic glands of the gastric fundus, accelerates gastric emptying [25] and elicits appetite [26] acting both at peripheral and central level. Therefore, circulating ghrelin concentration is high in fasting state and decreases in the postprandial period [27].

Previous literature focused on the dosage of ghrelin in patients with CeD, described a higher level of this peptide in CeD patients at diagnosis and a lower level in patients on a GFD [28,29], probably due to the changes in the nutritional state [30] or to the reduction of systemic inflammation after diagnosis [31].

In this frame, a gap of knowledge still exists in the literature on the GI peptide response to a meal in association to appetite sensations in CeD patients.

This study aimed to evaluate the post-prandial appetite sensations induced by a mixed meal in patients with CeD at diagnosis (CeDD) and on a GFD (CeDGF) and to clarify the role of GI hormone response.

To this purpose, a single day intervention study was conducted in coeliac and healthy subjects who consumed a test meal, recorded their appetite sensations and were subjected to blood drawings over 3 h after the meal. Plasma samples were analysed to monitor GI hormone response related to appetite and glucose metabolism.

## 2. Material & Methods

### 2.1. Subjects

Enrollment of CeD patients was carried out at the CeD outpatient clinic of the University of Salerno and the Federico II University of Naples. Eligible subjects were adult women, aged 18–40 years with normal weight (BMI 18.5–25 kg/m^2^) and with CeD, diagnosed according to the latest Guidelines from the British society of gastroenterology [1]. CeD diagnosis was based on the presence of GI symptoms, Marsh ≥ 2 histology and antigliadin antibodies up to the year 1999 (when the modern tests were not available) and from the year 2000 (when specific and sensitive serology became available) in the presence of Marsh ≥ 2 histology and with both anti-tissue transglutaminase (a-tTG) IgA > 7 U/mL and positive anti-endomysial (EMA). Patients were at diagnosis (CeDD), if they had received the CeD diagnosis over a month before the enrollment, and on a GFD (CeDGF) if they were on a GFD from at least 12 months.

A control group of healthy women was also recruited among the patients’ friends and the hospital staff. The inclusion criteria were the same as CeD patients but with the exclusion of the diagnosis of CeD, and any food allergy/intolerance and gastrointestinal disease.

For both groups of subjects (CeD and healthy), those with eating disorders evidenced by Three-Factor Eating Questionnaire [32] were excluded. Enrollment took place between May 2012 and June 2015. All subjects gave their written informed consent, and the Ethical Committee of the University of Salerno approved the study protocol.

### 2.2. Study Design

It was a one-day study based on a test meal. The day before the experimental session subjects were asked to avoid doing vigorous physical activity and to have a dinner consisting of a standardized meal including one portion of meat, one of cooked vegetable, two slices of bread (all type except wholegrain bread), and one piece of fruit, within 9 p.m. The maximum time of the dinner was fixed to have subjects fasting since at least 11 h and to avoid a metabolic effect of the time of previous meal on the test meal at the next day [33,34]. Similarly, subjects were recommended to avoid wholegrain wheat bread because it might attenuate the glycemic response at the subsequent meal (second meal effect) [35]. At dinner, subjects consumed the amount of allowed foods according to their actual appetite. Time of the dinner as well as types and amounts of foods consumed were recorded by subjects and referred to the experimenters on the next day. Indeed, on the experimental day, fasting subjects were invited to reach the laboratory at 8 a.m. by using car or bus, and were checked for the health condition, absence of menstruation, and compliance to the dinner recommendations. For those who did not satisfy this check, the experiment was postponed. The others had 10 min of resting before a cannula needle was put in their arm vein. After 5 min of resting, a first blood sample was collected (0 min, baseline). Then a test meal was offered and subjects were asked to consume it in 15 min. At 30 min, 60 min, 120 min and 180 min after food intake other blood samples were collected. At each time point and immediately before blood collection, subjects recorded their appetite sensations (including hunger, fullness and satiety) by visual analogue scale (VAS).

The nutritional composition of the meal is reported in Table 1. It included: 100 g of gluten-free bread, 50 g of ham, 30 g of cheese (stracchino type), 10 g of butter, yoghurt and one apple.

### 2.3. Blood Sample Collection and Preparation

For each time point, two different vacutainer^®^ tubes of 4 mL each were used to collect plasma samples. A protease inhibitor mix, consisting of dipeptidyl peptidase IV (DPP-IV) inhibitor (Millipore’s DPP-IV inhibitor; St Charles, MO, USA), protease inhibitor cocktail (Sigma, St. Louis, MO, USA) and 4-(2-Aminoethyl)-benzenesulfonyl fluoride hydrochloride (AEBSF, Pefabloc^®^ SC, Roche-diagnostics, Sigma, St. Louis, MO, USA), was immediately added only into the blood destined to the GI peptide. After sample preparation, tubes were centrifuged at 4000 g for 10 min and plasma samples were aliquoted and frozen at −80 °C, within 30 min from the collection.

Plasma samples were analysed for GI peptides concentration. Luminex Technology (Bio-Plex; Bio-Rad, Nazareth, Belgium) was used to determine GI peptides. A magnetic bead panel kit provided by Milliplex^®^ (Merck Millipore S.p.A., Darmstadt, Germany) allowed the simultaneous determination of the following four hormones: ghrelin, glucagon-like peptide-1 (GLP-1), glucose-dependent insulinotropic polypeptide (GIP), and insulin. The sensitivity levels of the assay (in pg/mL) were: ghrelin 2.0; GIP 0.6; GLP-1 7.0; insulin 58.0. The intra-assay coefficient of variation (%CV) was 2% for ghrelin, 3% for GIP and insulin; 7% for GLP-1. The inter-assay %CV was 5% for GIP; 6% for insulin; 8% for ghrelin; 10% for GLP-1.

### 2.4. Statistical Analysis

The analysis of variance (ANOVA) was performed to evaluate the differences between groups at each time points for both biochemical analysis and appetite scores. Since no difference at baseline for appetite scores was found, the results were analysed and expressed as the absolute changes from the baseline. The subjective appetite sensations, the glycaemia and the response of hormones were tested for the effect of time as a factor by the ANOVA for repeated measures. The linear trapezoidal rule was used to calculate the total area under the curves (AUC) for the appetite sensations and biochemical markers. Results are expressed as means ± SEM and were considered statistically significant for *p* < 0.05. Statistical analyses were performed using Statistical Package for Social Sciences (version 16.0; SPSS, Inc., Chicago, IL, USA).

## 3. Results

### 3.1. Subjects

Two eligible women were excluded, one because affected with an eating disorder, the second affected with type 1 diabetes. Twenty-two CeD patients (9 at diagnosis, CeDD, and 13 on a GFD, CeDGF) and 10 healthy subjects (HS) participated in the study. Table 2 reports their general characteristics (mean ± SD). The three groups were homogeneous for age (*p* = 0.09) and BMI (*p* = 0.3). Six subjects in the CeDGF group were on a GFD for more than 5 years in, 4 of them for 3 years, 2 subjects for 2 years, and 1 subject for one year.

### 3.2. Appetite

**Figure 1:**
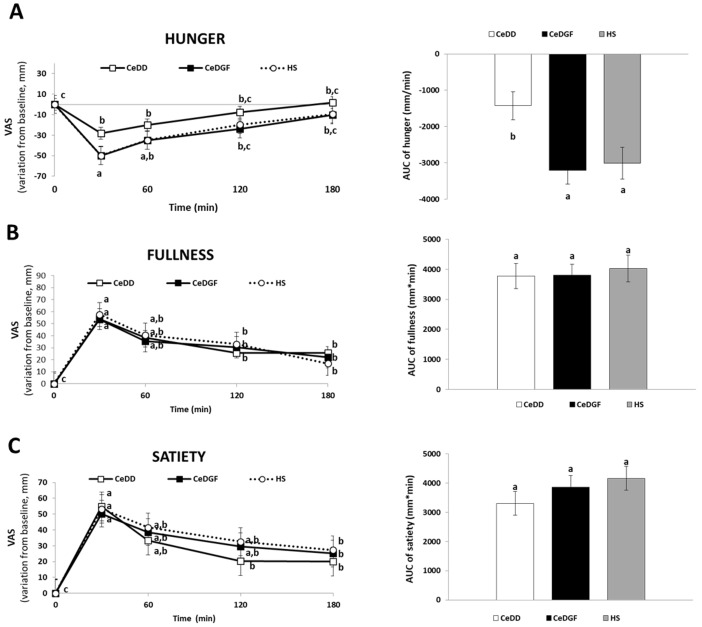
Post-prandial appetite. Variations from baseline and area under the curve (AUC) of hunger (**A**) fullness (**B**) and satiety (**C**) feelings recorded during the study. Different letters on appetite feelings’ lines indicate significant differences between times and groups; different letters on AUC bars indicate significant differences between groups (ANOVA and post-hoc Tukey test, *p* < 0.05).

No significant difference of hunger, fullness and satiety sensations at baseline between the groups was found. Figure 1 shows the variations from baseline of appetite ratings over time. Data showed a lower decrease in hunger in CeDD compared to CeDGF and HS after food intake.

### 3.3. Hormone Responses

**Figure 2:**
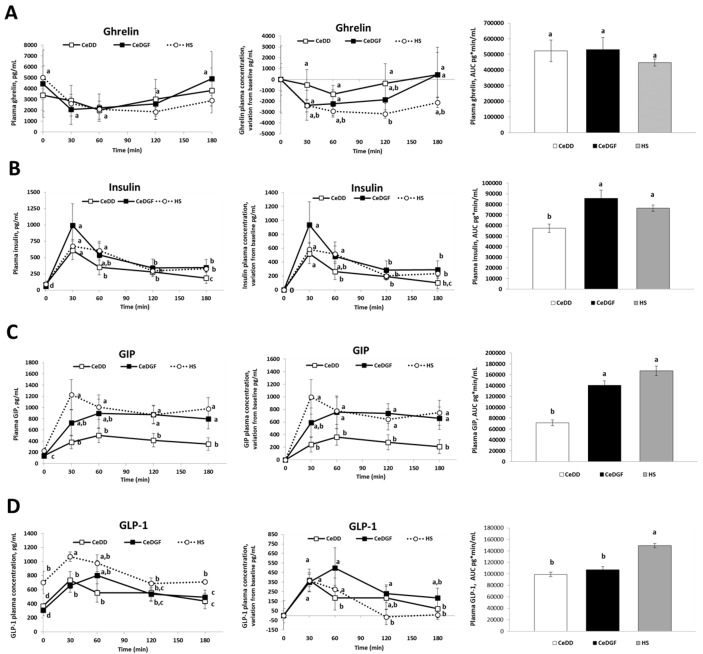
Post-prandial gastro-intestinal hormone response. Concentration-time response, variation from baseline and AUC of plasma concentration of ghrelin (**A**), insulin (**B**), GIP (**C**) and GLP-1 (**D**) over the study, into the three groups of subjects. Different letters on hormone plasma concentration lines indicate significant differences between times and groups; different letters on AUC bars indicate significant differences between groups (ANOVA and post-hoc Tukey test, *p* < 0.05).

Figure 2 shows the time-concentration curve, the variation from baseline and the area under the curve (AUC) of circulating ghrelin, insulin, GIP and GLP-1.

Data showed that CeDD tended to have lower reductions of ghrelin in the post-prandial phase than CeDGF and HS but a significant difference was recorded only at 120 min between CeDD and HS. The AUC of plasma ghrelin over the three hours post-lunch was not significantly different between groups. CeDD showed a lower response of insulin than CeDGF and HS. Patients with coeliac disease (both CeDD and CeDGF) showed a lower concentration of plasma GLP-1 at baseline and over the post-prandial phase than HS.

### 3.4. Blood Glucose

**Figure 3:**
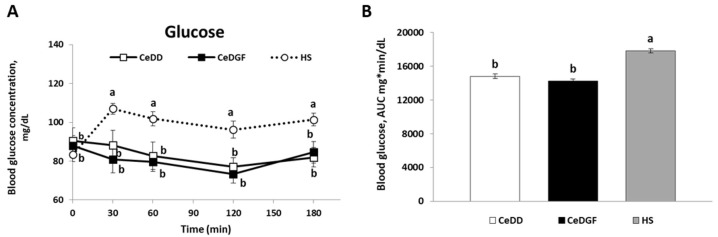
Post-prandial blood glucose. (**A**) The concentration-time response over three hours post-meal consumption in subjects with CeD at diagnosis (CeDD), on a GFD (CeDGF) and in healthy subjects (HS); (**B**) AUC of blood glucose over three hours post-meal in the three groups of subjects. Different letters on blood glucose concentration lines indicate significant differences between times and groups; different letters on AUC bars indicate significant differences between groups (ANOVA and post-hoc Tukey test, *p* < 0.05).

Figure 3 shows the blood glucose concentration-time curve over the study and the AUC of glucose in the three groups. Data showed that patients with coeliac disease (both CeDD and CeDGF) experienced lower post-prandial response of glucose than HS (*p* < 0.05).

## 4. Discussion

To the best of our knowledge, this is the first study investigating the post-prandial effect of a mixed gluten free meal on appetite sensations, blood glucose response and GI hormone response in patients with CeD at the diagnosis (before starting a GFD) and on a GFD (under a GFD since at least 12 months) in comparison with healthy subjects (HS).

The main finding of this study was that in the post-prandial phase CeDD showed a sustained hunger sensation and a reduced response of plasma GIP and insulin compared to CeDGF and HS. In CeDD, the lower hunger reduction could be explained by the lower GIP response than in CeDGF and HS, in association with the lower GLP-1 than HS. Indeed, GIP and GLP-1 delay gastric emptying at physiological concentration [36]. Therefore, a lower response of GIP in CeDD, in addition to the low GLP-1 level (as in CeDGF vs. HS), could determine a faster gastric emptying and, in turn, a faster returning of hunger. The ghrelin response recorded over time in the three groups were similar and confirmed the previous evidence that the delayed gastric emptying in CeD patients was not associated with reduced ghrelin levels [29].

Another important finding was the lower post-prandial glycaemia of patients with CeD, both CeDD and CeDGF, than HS. That finding was in agreement with the known condition for coeliac patients of a decreased glucose absorption as observed in an old study by measuring jejunal transmural potential differences [37] and as more recently demonstrated [38]. These authors reported in duodenal biopsies of active coeliac patients a reduced expression of the main solute transporters such as the Na+/glucose co-transporter 1 (SGLT1), the H+/oligopeptide transporter 1 (PEPT1) and Na+/H+ exchanger 3 (NHE3) thus revealing a clear mechanism underpinning impaired glucose, oligopeptide and sodium absorption in those patients. Moreover, they showed that tissue expression of the transporters was restored entirely in coeliac patients consuming a GFD for at least 12 months. Our data suggested that this was not the case for our subjects even if they were all under a GFD for a time longer than 12 months. However, the different hormonal response found into the two groups let us hypothesize the occurrence of concomitant mechanisms underpinning post-prandial glycaemia. In CeDD, additionally to a supposed impaired glucose absorption, the low glycaemia was coherently associated with low responses of the incretins GIP and GLP-1 as well as of insulin. Contrarily, CeDGF showed post-prandial insulin and GIP curves that were similar to HS. GIP can reduce postprandial blood glucose by prolonging insulin release or by improving glucose uptake directly in the liver or other peripheral tissues, as well as by inhibiting glucose output from the liver [23]. Therefore, supposing a similar absorption rate of glucose, hormonal data suggested that increased sensitivity of insulin and GIP in CeDD compared to CeDGF and HS might occur; this might explain the similar glucose curve of CeDD and CeDGF irrespective from GIP and insulin responses. In other words, GIP and insulin were more effective in glucose uptake at the cellular level in CeDD than CeDGF. Such a phenomenon may be an adaptive physiological response of the body to the condition of malabsorption typical of CeDD and is coherent with mechanisms occurring after weight loss [39].

Interestingly, patients with coeliac disease (both CeDD and CeDGF) showed a lower blood GLP-1 concentration than HS at every time point as previously shown in fasting children with CeD [40]. Since no difference for post-prandial blood glucose between CeDD and CeDGF was found, similar circulating level of GLP-1 in the two groups supported a central role of this peptide in glucose management as well as the hypothesis (above discussed) of a similar expression/activity of SGLT-1 in CeDD and CeDGF in our study [41].

The hypercaloric/iperlipidic content of commercially available gluten free foods [42] may cause the reported weight gain and frequency of obesity of CeDGF [43] with increase of cardiovascular risk.

The present findings add a piece of information to the understanding of the post-prandial response to a GF meal that may contribute to the nutritional and metabolic consequences of a GFD in the long period.

## 5. Conclusions

This study shows the evolution of appetite sensations, blood glucose and the GI peptide response in CeD patients both on a gluten-containing diet and on GFD in comparison with HS over three hours after the consumption of a gluten free meal.

Main findings showed that CeDD experienced a sustained hunger sensation and a reduced response of plasma GIP and insulin compared to CeDGF and HS and coeliac patients (both CeDD and CeDGF) showed a lower post-prandial glycaemia and plasma GLP-1 than HS.

Overall data suggested that CeD subjects after more than one year of a GFD did not recover a complete functionality of the intestinal hormone response to a meal. We hypothesize that the described alteration might determine an abnormal adaptive hormone postprandial response that could influence post-prandial appetite sensations and insulin resistance over some time after diagnosis, thus contributing to the weight gain. However, we cannot exclude that the presence of sustained hunger sensation will disappear during long-lasting GFD.

## Figures and Tables

**Table 1:** Nutritional composition of the meal test.

Meal Test	Protein (g)	Fat (g)	SFA (g)	MUFA (g)	PUFA (g)	CHO (g)	Fiber (g)	Energy (kcal)
**Gluten free bread 100 g**	3.2	2.5	0.4	0	0	45.3	6.3	229
**Ham 50 g**	13.4	1.6	0.5	0.1	0.1	0	0	68
**Cheese 30 g**	5.6	7.5	4.7	2.4	0.3	0	0	90
**Butter 10 g**	0.1	8.3	4.9	2.4	0.3	0.1	0	75.8
**Yogurt 125 g**	4.8	4.9	2.6	1.1	0.2	5.4	0	82.5
**Apple 150 g**	0.5	0.2	0	0	0	20.6	3	79.5
**Total**	**27.4**	**25.0**	**13.1**	**5.99**	**0.78**	**71.3**	**9.30**	**624.8**

SFA: saturated fatty acids; MUFA: monounsaturated fatty acids; PUFA: polyunsaturated fatty acids; CHO: carbohydrates.

**Table 2:** Characteristics of study participants.

	CeDD	CeDGF	HS	*p* Value
**Age (years)**	29.4 ± 2.3	30.6 ± 3.6	31.6 ± 4.0	0.1
**BMI (kg/m^2^)**	21.9 ± 20.1	22.2 ± 1.9	22.0 ± 1.9	0.3
**a-tTG (U/mL)**	142.8 ± 28.2	2.84 ± 0.02	−	<0.001

CeDD: coeliac disease patients at diagnosis; CeDGF: coeliac disease patients on a gluten-free diet; HS: healthy subjects. Data was reported as mean ± standard deviation.
